# Assessing the utility of molecular diagnostic classification for cancers of unknown primary

**DOI:** 10.1002/cam4.6532

**Published:** 2023-09-15

**Authors:** Elle C. Moore, Gerard C. Blobe, Nicholas C. DeVito, Brent A. Hanks, Michael R. Harrison, Christopher J. Hoimes, Jingquan Jia, Michael A. Morse, Parvathy Jayaprakasan, Andrew MacKelfresh, Hillary Mulder, Adam J. Hockenberry, Alia Zander, Martin C. Stumpe, Jackson Michuda, Kyle A. Beauchamp, Eric Perakslis, Timothy Taxter, Daniel J. George

**Affiliations:** ^1^ Tempus Labs, Inc Chicago Illinois USA; ^2^ Division of Medical Oncology, Department of Medicine Duke University School of Medicine Durham North Carolina USA; ^3^ Department of Pharmacology and Cancer Biology Duke University Medical Center Durham North Carolina USA; ^4^ Center for Cancer Immunotherapy Duke University Medical Center Durham North Carolina USA; ^5^ Duke Cancer Institute Center for Prostate and Urologic Cancers Durham North Carolina USA; ^6^ Duke Clinical Research Institute Duke University Medical Center Durham North Carolina USA

## Abstract

**Background:**

Roughly 5% of metastatic cancers present with uncertain origin, for which molecular classification could influence subsequent management; however, prior studies of molecular diagnostic classifiers have reported mixed results with regard to clinical impact. In this retrospective study, we evaluated the utility of a novel molecular diagnostic classifier by assessing theoretical changes in treatment and additional testing recommendations from oncologists before and after the review of classifier predictions.

**Methods:**

We retrospectively analyzed de‐identified records from 289 patients with a consensus diagnosis of cancer of uncertain/unknown primary (CUP). Two (or three, if adjudication was required) independent oncologists separately reviewed patient clinical information to determine the course of treatment before they reviewed results from the molecular diagnostic classifier and subsequently evaluated whether the predicted diagnosis would alter their treatment plan.

**Results:**

Results from the molecular diagnostic classifier changed the consensus oncologist‐reported treatment recommendations for 235 out of 289 patients (81.3%). At the level of individual oncologist reviews (*n* = 414), 64.7% (*n* = 268) of treatment recommendations were based on CUP guidelines prior to review of results from the molecular diagnostic classifier. After seeing classifier results, 98.1% (*n* = 207) of the reviews, where treatment was specified (*n* = 211), were guided by the tissue of origin‐specific guidelines. Overall, 89.9% of the 414 total reviews either expressed strong agreement (*n* = 242) or agreement (*n* = 130) that the molecular diagnostic classifier result increased confidence in selecting the most appropriate treatment regimen.

**Conclusions:**

A retrospective review of CUP cases demonstrates that a novel molecular diagnostic classifier could affect treatment in the majority of patients, supporting its clinical utility. Further studies are needed to prospectively evaluate whether the use of molecular diagnostic classifiers improves clinical outcomes in CUP patients.

## 1 INTRODUCTION

Cancer of unknown primary (CUP) describes a heterogeneous group of metastatic tumors that are defined by the absence of a clinically known tissue of primary origin. Patients diagnosed with CUP represent 2%–5% of all cancers,^1^, ^2^ and treatment options available to CUP patients are limited.^3^, ^4^ Compared to patients with known primary tumors, the prognosis for CUP patients is generally poor, with median overall survival rates reported as <12 months.^2^, ^5^, ^6^

In the absence of a known primary site, clinicians gather information from a range of sources—including imaging and endoscopy as well as histopathological, molecular, and serum biomarkers—to aid in treatment selection.^6^, ^7^ More recently, comprehensive genomic profiling using next‐generation sequencing (NGS) has been used to identify tumor‐specific mutations to inform targeted treatment options.^1^ Numerous assays that rely on either genomic profiling, microRNA expression, DNA methylation, transcriptome expression, or some combination of these data sources have been developed to aid in diagnosing the tissue of origin for CUP patients.^8^, ^9^, ^10^ The Tempus Tumor Origin (TO) test uses RNA expression data to select from 68 possible histological subtypes and showed a 91% classification accuracy when applied to an independent validation set of samples with known primary.^11^

A limited number of studies have evaluated the clinical utility of tissue‐of‐origin diagnosis via molecular diagnostic classifier in the setting of CUP owing to methodological challenges with studying this population.^12^ Several randomized studies have found no significant difference in CUP patient outcomes according to whether they received tissue‐specific or empiric therapies,^13^, ^14^ while another showed that patients receiving tissue‐specific therapies have improved outcomes.^15^ Prior studies evaluating the impact of identifying a primary tissue of origin have either focused on investigating outcomes within specific predicted subtypes^16^, ^17^ or replicating known prognostic differences among subtypes.^13^, ^18^, ^19^, ^20^ Despite limitations, results from these studies indicate potential clinical benefit of having a diagnosis assigned by a molecular diagnostic classifier.

Current NCCN treatment guidelines focus on the tissue of origin for recommendation (i.e., breast cancer),^21^ suggesting that a molecular‐guided diagnosis for CUP patients could alter therapeutic management. Toward this end, a prospective study from 2018 found that 47% of patients had an altered treatment plan following review of tissue‐of‐origin predictions from a 92‐gene assay.^22^ However, this study was limited by the inclusion of a considerable number of patients with a suspected site of origin and few predicted tumor (sub‐)types in the population; in addition, since 2018, treatment paradigms based on genomic profiling have changed dramatically. Here, we present a decision impact study where oncologists retrospectively reviewed redacted clinical records from 289 confirmed CUP patients. From these reviews, we assessed treatment and testing recommendations both prior to and after review of molecular diagnostic classifier results, as well as reported confidence that the classifier results aided in selecting the most appropriate treatment regimen.

## 2 METHODS

### 2.1 Study population

An initial set of 336 de‐identified records from patients with a CUP diagnosis whose clinician ordered Tempus xT (CAP/CLIA validated DNA‐seq of 648 genes)^23^, ^24^ and the Tempus Tumor Origin (TO) test (v.1.0 for reports before 10/28/2021, v.1.1 for subsequent orders) were selected by simple random sampling (each case was randomly assigned an integer and a random number generator selected a subset of integers) from the full set of 519 patients meeting all eligibility criteria in the Tempus clinical‐genomic database. These patients came from 169 different clinical sites across the United States. At least 285 patients were required based on statistical power requirements of 80% power at an alpha of 0.05 to test the null hypothesis of treatment change exceeding 40% and a non‐inferiority difference of 0.07. A total of 336 patients were included based on the expectation that approximately 10% of patients would be screened out of the study due to not having a confirmed CUP diagnosis.

The classification of CUP in the Tempus clinical‐genomic database is established by Tempus pathologists who review pathology reports and prognosis notes as part of routine sample accessioning and report signout (cancer diagnoses are used to guide therapy and clinical trial matching on the xT report). When a definitive diagnosis is not provided in the associated reports the patient is classified as CUP (i.e., Tempus pathology does not assign speculative diagnoses when the clinical care team is considering multiple diagnoses). Each eligible patient had a TO result issued over a period in 2021, a predicted probability for one or more diagnoses generated by the TO test, an ECOG score ≤2, and at least one pathology report (inclusive of any available diagnostic IHC, microscopic findings, any relevant addendums, etc.) and one progress note (inclusive of any available relevant patient and familial history, lab results [especially blood tumor markers], radiology, etc.) from the time of testing on file.

### 2.2 Review of clinical records and treatment assignment

Each patient record was randomly assigned to be initially reviewed by two independent board‐certified oncologists from an NCI‐designated Comprehensive Cancer Center, with sub‐specialties including gastrointestinal cancers, genitourinary cancers, neuroendocrine cancers, and cancers of unknown primary. The initial set of two reviews for each patient were performed by seven different oncologists in total. We utilized a case assignment method that aimed to achieve equal overlap between each reviewer and their counterparts, where a given reviewer shared an equal number of cases with each of the other reviewers. In the event of disagreement about whether the patient was CUP or on the primary endpoint (a yes or no question asking whether the results from the molecular diagnostic classifier would change treatment recommendations), a third oncologist reviewer adjudicated the discordance. These reviews were randomly assigned to one of three oncologists, and the adjudication reviewer never adjudicated a set of reviews that they initially reviewed.

For the determination of CUP status and inclusion in the study, reviewing oncologists used clinical and pathologic data available in progress notes from when the molecular diagnostic classifier was ordered, pathology reports, and NGS results (including tumor mutational burden, microsatellite instability status, and DNA mutational status from any of the available 648 genes on the panel including fusions). The reviewing oncologists provided their responses in a standardized questionnaire template (see Data S1 for a full list of all questions that reviewers were asked). Specifically for confirmation of CUP status, reviewing oncologists were asked “Does the patient's cancer appear to be of uncertain/unknown primary?” and required to pick either: (1) Yes, (2) No, or (3) Not enough information. In the event that the available clinicopathologic information and NGS results met criteria for assigning a primary diagnosis, the reviewer designated the case as non‐CUP, and the patient was excluded from the study. Only patients with a consensus answer of “yes”—indicating they are definitionally CUP—were included in the study.

For assessment of the primary endpoint, the reviewing oncologists specified the patient's treatment regimen before and after receiving the molecular diagnostic classifier result in free text, while having the option to leave responses blank if they felt there was not enough information. For reviews where additional testing was not needed, they were then asked “Did the treatment regimen change?” and had to pick one of the following answer options: (1) Yes, the entire regimen was updated, (2) Yes, I added an agent, (3) Yes, I removed an agent, (4) No, (5) N/A–Not enough information to select a treatment, or (6) Other (opens free text). If additional testing was needed, the reviewer answered “How would the results of additional organ‐specific testing change treatment?” and selected: (A) Treatment would change regardless of the results of testing, (B). Treatment change is dependent on result of testing, (C)Treatment would not change, or (D) Other (opens free text). The answers that counted as “yes” toward the primary endpoint of whether treatment would change included selection of either 1, 2, 3, A, or B. If there was disagreement regarding whether treatment would have changed between the initial two reviewing oncologists, a third oncologist adjudicated the result to allow the primary endpoint to be assessed at the patient level.

In addition to providing treatment recommendations pre‐ and post‐review of the molecular diagnostic classifier results, oncologists were also asked several follow‐up questions regarding the need for additional diagnostic testing, the evidence that guided treatment recommendations, and confidence that the molecular diagnostic classifier results aided in selecting the most appropriate treatment regimen. Any differences in secondary endpoints or follow‐up questions (including specific treatment recommendations at any stage of review) were not adjudicated.

### 2.3 Data collection and statistical analyses

Standardized patient study assessment forms were filled out electronically by oncologists before and after reviewing the results of the molecular diagnostic classifier. Structured fields and free text results associated with medications and regimens were curated and aggregated for study analysis. Descriptive study analysis was performed according to SAP v1.0 using SAS version 9.4 (SAS Institute Inc.).

Patient characteristics were summarized as counts (%) for categorical variables and mean (standard deviation) and median (25th, 75th percentiles) for continuous variables. The primary endpoint was presented as a binomial proportion with 95% confidence intervals calculated using the Wilson score method without continuity correction. Results of the oncologist surveys were summarized on the review or patient level according to whether the question was adjudicated.

### 2.4 Molecular diagnostic classifier description

The Tempus TO test is a CAP/CLIA validated molecular diagnostic classifier that uses whole‐exome capture RNA‐seq data as input to provide diagnostic predictions from 68 possible subtypes. The model was trained on 43,726 tumor samples of known origin and has an overall accuracy of 91% based on an independent validation cohort consisting of 9210 tumor samples.^11^ For individual patient records, results are reported with one or more predicted diagnoses including the probability that the sample belongs to the given label. Samples where the highest‐probability label is lower than the 30% (TO v1.0) or 35% (TO v1.1) threshold are reported with an “indeterminate” result. The differences between the v1.0 and v1.1 versions of the TO test are minor and not expected to impact the results of the study.

## 3 RESULTS

### 3.1 Study overview and clinical characteristics

We collected de‐identified records from patients with confirmed cancers of unknown primary—defined here as a patient with two or more possible sites of tumor origin. Treatment recommendations and confirmation of CUP status were made by oncologist reviewers based on review of all available clinical information (including molecular NGS reports) before viewing predictions from the molecular diagnostic classifier. In the event that the reviewer felt there was not enough information available to select an initial treatment, they were not required to select one. After reviewing the classifier results, oncologists again reported treatment recommendations. Each patient record was independently assessed by two oncologists, with any differences in the eligibility (whether the patient had a CUP) or primary endpoint (a yes/no response regarding whether results from the molecular diagnostic classifier produced a change in treatment recommendations) adjudicated by a third oncologist. Data S1 lists the full set of questions that oncologists were asked to fill out for each patient.

**FIGURE 1:**
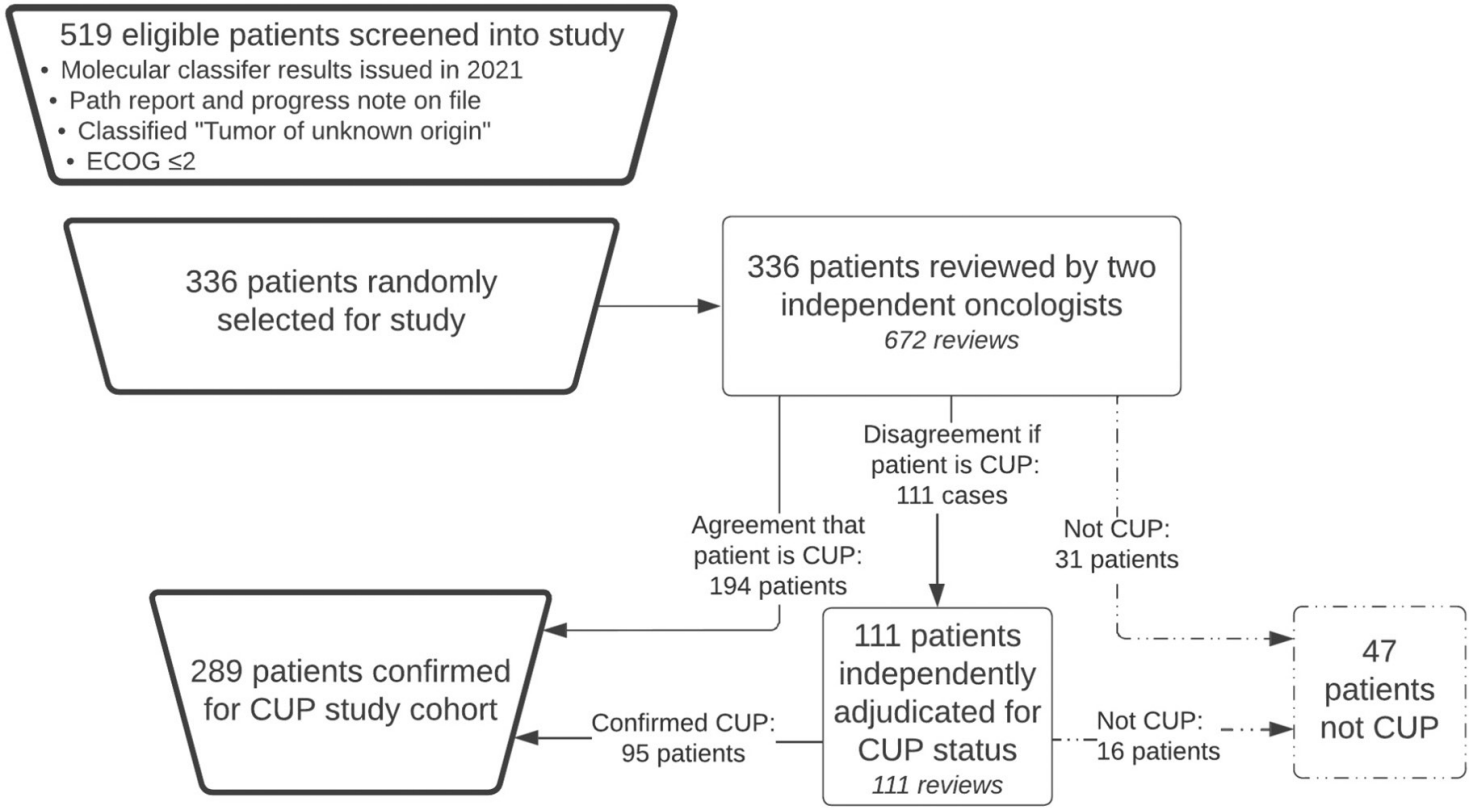
Overview of patient inclusion criteria and reviewer confirmation of cancer of uncertain/unknown primary (CUP) status.

Following review of available clinical information, 47 patients with a cancer classified as “tumor of unknown origin” did not meet the study inclusion criteria. For 31 of these patients, both initial reviewers agreed that the cancer was not CUP, and for 16 patients, a third reviewer confirmed that the cancer was not CUP. Figure 1 shows the flow of patients with regard to screening and eligibility. Our study thus assessed 289 patients with a consensus diagnosis of CUP, and of these, 111 (38.4%) required adjudication based on initial disagreement about the CUP diagnosis. An additional set of patients (*n* = 53, 18.3% of the assessed patient population) required adjudication due to disagreement regarding whether treatment would change following review of the molecular diagnostic classifier results.

The most common predicted subtypes reported by the molecular diagnostic classifier were: cholangiocarcinoma (*n* = 58, 20.1%), lung adenocarcinoma (*n* = 41, 14.2%), pancreatic adenocarcinoma (*n* = 22, 7.6%), lung squamous cell carcinoma (*n* = 19, 6.6%), colorectal adenocarcinoma (*n* = 18, 6.2%), and gastroesophageal adenocarcinoma (*n* = 18, 6.2%). Table 1 lists patient and tumor characteristics including biopsy site and demographics, as well as a summary of results from the molecular diagnostic classifier.

**TABLE 1:** Patient demographics and molecular diagnostic classifier results.

Characteristic	CUP patients (*n* = 289)
Age (years)	
Mean (SD)	66.1 (11.3)
Median (Q1, Q3)	66.0 (59.0, 73.0)
Female	145 (50.2%)
Biopsy site	
Abdomen	54 (18.7%)
Axilla	6 (2.1%)
Bone and soft tissue	28 (9.7%)
Brain	10 (3.5%)
Breast	1 (0.3%)
GI tract	12 (4.2%)
GU	3 (1.0%)
Gynecologic	7 (2.4%)
Head and neck	22 (7.6%)
Liver	87 (30.1%)
Lung	22 (7.6%)
Pelvis	10 (3.5%)
Skin	6 (2.1%)
Thorax	21 (7.3%)
Molecular diagnostic classifier predicted primary diagnosis probability	
*N*	289
Mean (SD)	77.1% (20.6)
Median (Q1, Q3)	84.0% (61.0%, 95.0%)
Molecular diagnostic classifier predicted diagnosis	
Adenoid cystic carcinoma	1 (0.3%)
Adrenal cortical carcinoma	1 (0.3%)
Anogenital squamous cell carcinoma	4 (1.4%)
Breast carcinoma	12 (4.2%)
Carcinosarcoma	5 (1.7%)
Cervical carcinoma	1 (0.3%)
Cholangiocarcinoma	58 (20.1%)
Colorectal adenocarcinoma	18 (6.2%)
Endometrial endometrioid carcinoma	2 (0.7%)
Endometrial serous carcinoma	1 (0.3%)
Endometrioid carcinoma	1 (0.3%)
Fibrous sarcoma	1 (0.3%)
Gastroesophageal adenocarcinoma	18 (6.2%)
Gastroesophageal squamous cell carcinoma	4 (1.4%)
Goblet cell adenocarcinoma	1 (0.3%)
Head and neck squamous cell carcinoma	7 (2.4%)
Hepatocellular carcinoma	3 (1.0%)
Leiomyosarcoma	1 (0.3%)
Liposarcoma	2 (0.7%)
Lung adenocarcinoma	41 (14.2%)
Lung squamous cell carcinoma	19 (6.6%)
Melanoma	3 (1.0%)
Ovarian clear cell carcinoma	1 (0.3%)
Ovarian mucinous adenocarcinoma	2 (0.7%)
Ovarian serous carcinoma	11 (3.8%)
Pancreatic adenocarcinoma	22 (7.6%)
Pancreatic neuroendocrine carcinoma	2 (0.7%)
Peripheral nerve sheath tumor	1 (0.3%)
Prostate neuroendocrine carcinoma	1 (0.3%)
Prostatic adenocarcinoma	2 (0.7%)
Renal chromophobe carcinoma	1 (0.3%)
Renal clear cell carcinoma	2 (0.7%)
Renal papillary carcinoma	1 (0.3%)
Salivary carcinoma	5 (1.7%)
Skin neuroendocrine carcinoma	1 (0.3%)
Skin squamous and basal cell carcinoma	4 (1.4%)
Small bowel adenocarcinoma	1 (0.3%)
Small bowel and appendiceal adenocarcinoma	3 (1.0%)
Small cell lung carcinoma	9 (3.1%)
Thymic squamous cell carcinoma	1 (0.3%)
Thyroid cancers	2 (0.7%)
Urothelial carcinoma	13 (4.5%)

### 3.2 Treatment recommendations before and after review of molecular diagnostic classifier results

**FIGURE 2:**
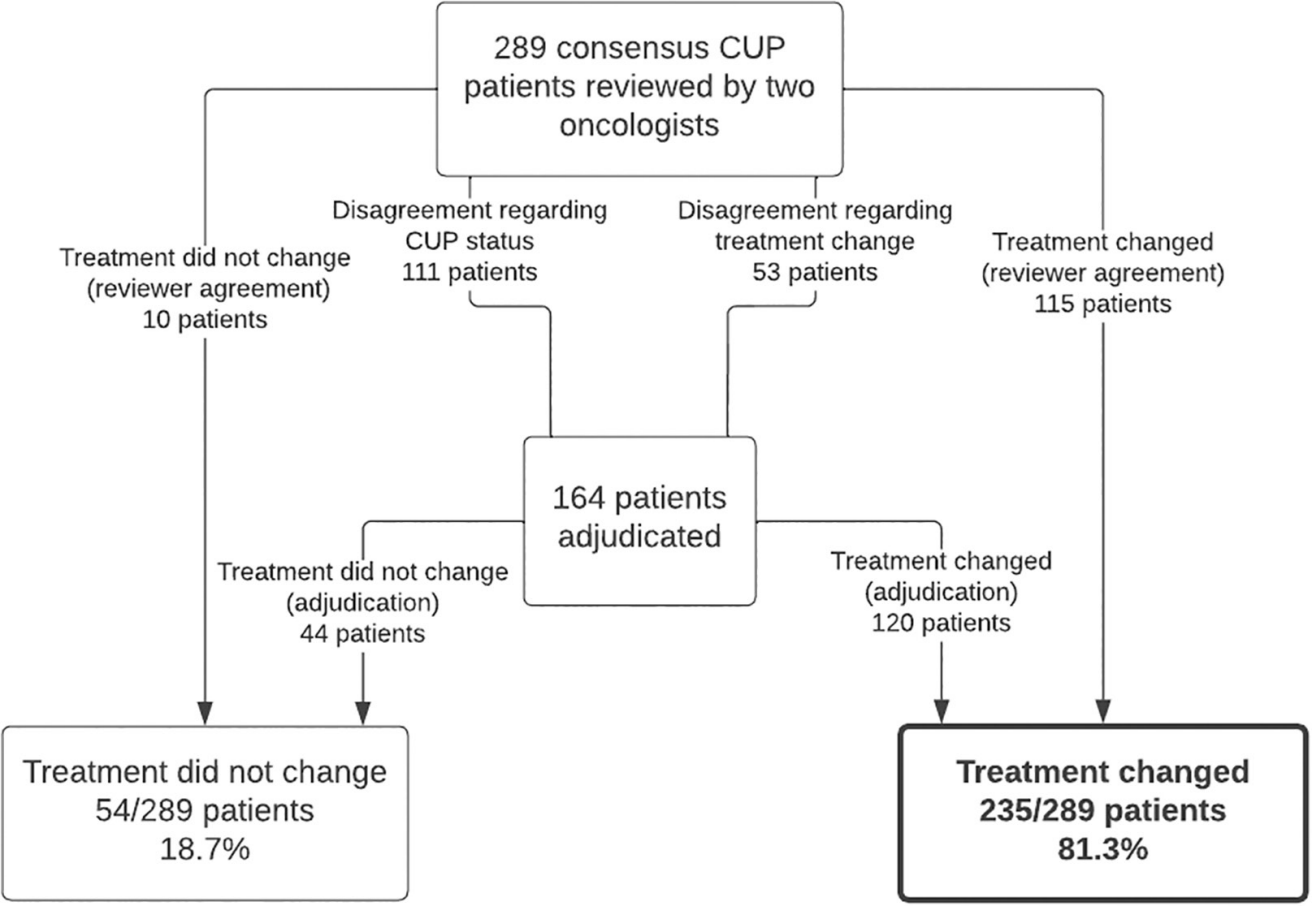
Overview of patient‐level findings with regard to the primary study endpoint (oncologist assessment of treatment change based on molecular diagnostic classifier results).

Of the 289 patients analyzed in this study, the reviewing oncologists indicated that 81.3% (*n* = 235, 95% confidence interval = [76.4, 85.4]) of patients would have some form of change in their treatment plan based on the results from the molecular diagnostic classifier—Either specifying a treatment where there was previously not enough information available to do so, addition/removal of a treatment agent, complete update of the treatment regimen, or based on additional biomarker testing. Figure 2 shows that for the 125 patients that did not require adjudication of the primary endpoint (i.e., there was consensus from the two reviewers as to whether the treatment would/would not change following review of classifier results as well as on the initial CUP diagnosis), reviewers agreed that treatment recommendations changed for 92.0% (*n* = 115) and did not change for 8.0% (*n* = 10) of patients. For the 164 patients that required adjudication from a third oncologist, the adjudicating reviewer indicated that treatment changed for 73.2% (*n* = 120) and did not change for 26.8% (*n* = 44) of patients. Considering only the subset of patients where the assessed review/s contained a selected treatment prior to review of the molecular diagnostic classifier (*n* = 222), reviewing oncologists indicated that 75.7% (*n* = 168) would have some form of a change in their treatment plan. Finally, to assess possible reviewer heterogeneity, we looked at data from individual reviewers (8 reviewers, each reviewing between 9 and 90 patient records) and found that the percentage of cases where reviewers stated that treatment recommendations would change following review of classifier results ranged from 73%–97%.

**FIGURE 3:**
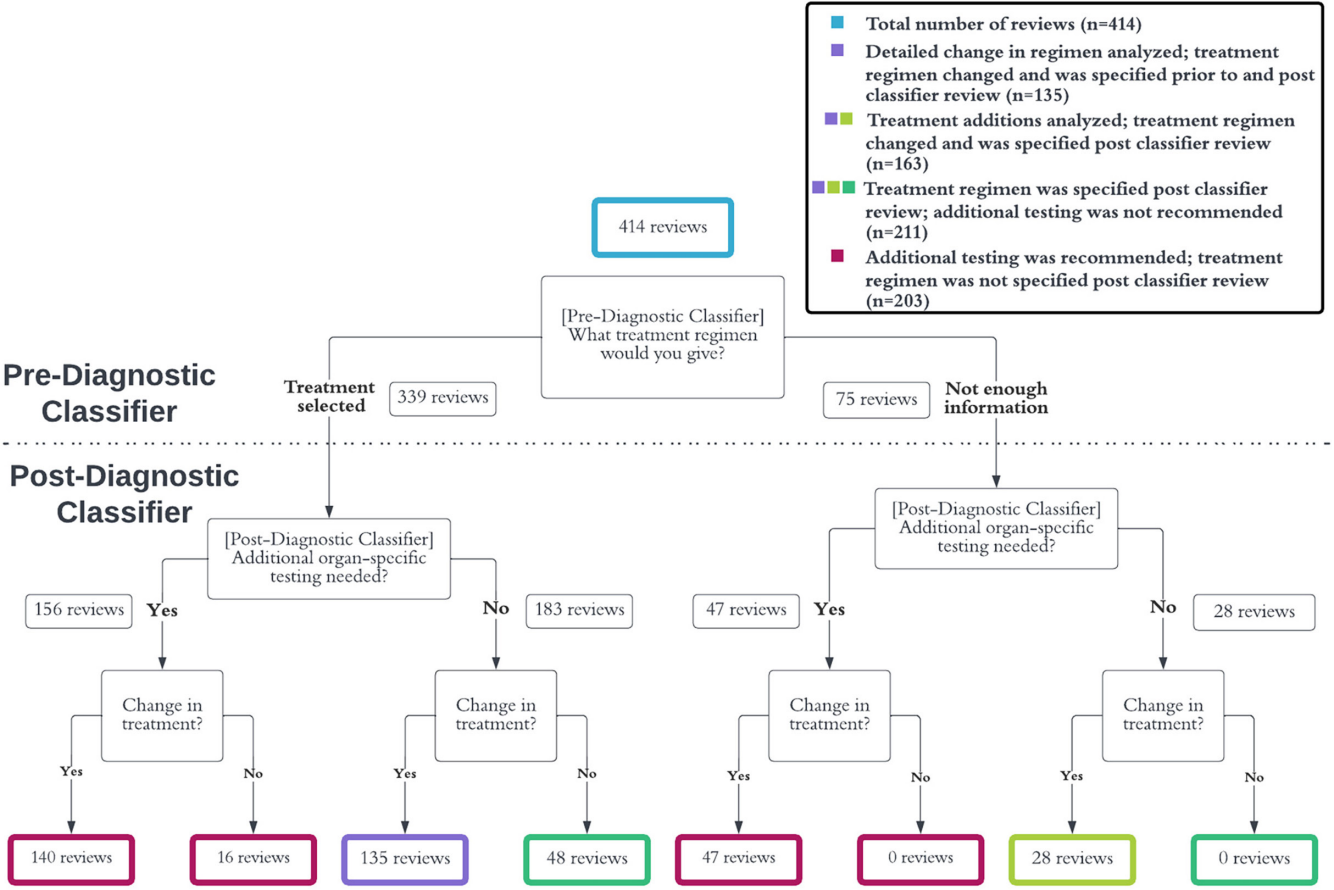
Overview of individual reviews split according to responses to clinical management questions. The number of reviews analyzed for certain secondary endpoints—including characterizing therapy changes and the evidence guiding treatment regimen determination—is dependent on answers to these questions. Of the questions shown, only “Change in treatment?” was adjudicated in the event of disagreement by the initial reviewers.

Consensus patient‐level results were used for the aforementioned primary endpoint because responses to this line of inquiry were adjudicated. By contrast, subsequent analyses with regard to questions that were not adjudicated were performed on the reviews from individual oncologists. In total, there were thus 125 patient records where the initial two reviewers agreed on both the CUP diagnosis as well as the primary endpoint (resulting in 250 reviews). There were 164 patient records confirmed CUP through adjudication where the first two reviewers disagreed on either CUP status (*n* = 111) or primary endpoint (*n* = 53), necessitating adjudication by a third reviewer who summarized and resolved conflicting information from the first two reviewers. For these adjudicated patients, the third review was regarded as the consensus review and was subsequently assessed in review‐level analyses. Thus, the 289 patient records were associated with 414 reviews (250 + 164). Figure 3 represents the assessment flow and the number of reviews included in each subanalysis that follows.

Prior to review of the classifier results, oncologists characterized the type of treatment regimen (*n* = 339, 81.9%) or indicated there was not enough information available to select a treatment (*n* = 75, 18.1%; Table 2). Treatment recommendations from these individual reviews (*n* = 414) were most commonly based on CUP guidelines (*n* = 268, 64.7% of reviews) and a presumed primary site (*n* = 55, 13.3% of reviews). Following review of the classifier results, the oncologist could again specify a treatment or indicate that additional testing would be needed to specify a treatment. Of the 211 reviews where treatment was specified post‐classifier review, 98.1% (*n* = 207) were guided by the tissue of origin‐specific guidelines.

**TABLE 2:** Change in treatment regimen and feedback after review of molecular diagnostic classifier results (reviewer level).

	CUP reviews (*n* = 414)
Type of treatment, prior to molecular diagnostic classifier review (non‐mutually exclusive)	
CUP guidelines‐guided	268/414 (64.7%)
Molecular‐guided	33/414 (8.0%)
Presumed primary/primaries‐guided	55/414 (13.3%)
Histology‐guided	4/414 (1.0%)
Would not treat	1/414 (0.2%)
N/A–not enough information to select a treatment	75/414 (18.1%)
Type of treatment, post molecular diagnostic classifier for reviews where additional testing was not needed (non‐mutually exclusive)	
CUP guidelines‐guided	2/211 (0.9%)
Molecular‐guided	11/211 (5.2%)
Organ of origin‐specific	207/211 (98.1%)
Additional organ‐specific testing needed to make a treatment decision (non‐mutually exclusive)	
PD‐L1	118/414 (28.5%)
HRD	19/414 (4.6%)
ER/PR/HER2	31/414 (7.5%)
Additional imaging	60/414 (14.5%)
Other	55/414 (13.3%)
None requested	211/414 (51.0%)
How would the results of additional organ‐specific testing change treatment?	
Treatment would change regardless of the results of testing	62/203 (30.5%)
Treatment change is dependent on result of testing	125/203 (61.6%)
Treatment would not change	16/203 (7.9%)
Additional testing not requested	
Any change in treatment regimen	163/211 (77.3%)
Yes, the entire regimen was updated	143/163 (87.7%)
Yes, I added an agent	18/163 (11.0%)
Yes, I removed an agent	2/163 (1.2%)
No	48/211 (22.7%)
N/A–not enough information to select a treatment	0/211 (0.0%)
Order of confirmatory germline testing (non‐mutually exclusive)	
Yes	105/414 (25.4%)
Yes, based on the TO test results	52/105 (49.5%)
Yes, based on family history	58/105 (55.2%)
Yes, based on previous germline testing	11/105 (10.5%)
Yes, based on patient history of cancer	1/105 (1.0%)
Yes, based on NGS testing results	4/105 (3.8%)
No	300/414 (72.5%)
Not enough information to determine	9/414 (2.2%)
The TO result increased my confidence in selecting the most appropriate treatment regimen	
Strongly agree	242/414 (58.5%)
Agree	130/414 (31.4%)
Neutral	31/414 (7.5%)
Disagree	10/414 (2.4%)
Strongly disagree	1/414 (0.2%)

Of the 163 reviews where review of the molecular diagnostic classifier results led to an altered or new treatment plan without additional testing required (*n* = 135 reviews where a previous treatment was specified and *n* = 28 where no previous treatment was specified), the treatment recommendations most commonly added were gemcitabine (*n* = 69, 42.3%) and cisplatin (*n* = 62, 38.0%; Table 3). Other notable therapeutic changes following review of the classifier results were that checkpoint inhibitor therapy was added to 16.0% of reviews (*n* = 26) and a TKI was added to 8.0% of reviews (*n* = 13).

**TABLE 3:** Alterations to therapeutic management—The most common/clinically relevant added therapies, therapy changes, and removed therapies.

	Assessed reviews
Added therapies (treatment was specified post‐classifier review)	
Gemcitabine	69/163 (42.3%)
Cisplatin	62/163 (38.0%)
Checkpoint inhibitor (CPI) immunotherapy	26/163 (16.0%)
Tyrosine kinase inhibitor	13/163 (8.0%)
Therapy changes (treatment was specified prior to and post‐classifier review)	
Updated chemo	87/135 (64.4%)
Removed chemo, CPI only	8/135 (5.9%)
Systemic to local	4/135 (3.0%)
Removed therapies (treatment was specified prior to and post‐classifier review)	
Fluorouracil	46/135 (34.1%)
Oxaliplatin	42/135 (31.1%)
Carboplatin	41/135 (30.4%)
Paclitaxel	37/135 (27.4%)

In 135 reviews, a treatment regimen was specified prior to molecular diagnostic classifier review (i.e., there was sufficient information to determine a treatment plan based on other available clinical information) but subsequently updated post‐classifier review (with no additional testing needed). These reviews represent potentially the most impactful shifts in treatment recommendations in response to the results of the molecular diagnostic classifier, given that enough information was present to specify a treatment prior to review of the classifier results but that treatment regimen was subsequently changed without the need for additional testing. In these reviews, the treatments that were most commonly removed from recommended patient treatment plans following the review of results from the molecular diagnostic classifier were: fluorouracil (*n* = 46, 34.1%), oxaliplatin (*n* = 42, 31.1%), carboplatin (*n* = 41, 30.4%), and paclitaxel (*n* = 37, 27.4%; Table 3). When characterizing the types of changes made to therapy recommendations, 64.4% of changes (*n* = 87) were from one chemotherapy regimen to another, and 5.9% of changes (*n* = 8) were due to the removal of all chemotherapy agents and switch to immune checkpoint inhibitors only (CPIs). Further, 3.0% of patients (*n* = 4) were switched from systemic to local therapies.

### 3.3 Subtype‐specific biomarker and other additional testing recommendations

For the 49.0% of reviews (*n* = 203) where some form of additional testing was recommended, oncologists reported that treatment would change as a result of these tests in 92.1% (*n* = 187) of reviews (Figure 3). A majority of the additional testing recommendations were for assessment of biomarkers that are required for pre‐treatment evaluation in the guidelines corresponding to the cancer type predicted by the molecular diagnostic classifier. The types of additional testing most often specified as being needed to make a treatment decision were PD‐L1 (*n* = 118, 58.1%), additional imaging (*n* = 60, 29.6%), ER/PR/HER2 (*n* = 31, 15.3%), and HRD (*n* = 19, 9.4%), among others. The predicted subtypes with the greatest proportion of reviews requiring additional testing (with a minimum of 5 reviews) were gastroesophageal adenocarcinoma (20/24 reviews, 83.3%), gastroesophageal squamous cell carcinoma (5/6 reviews, 83.3%), head and neck squamous cell carcinoma (9/11 reviews, 81.8%), and breast carcinoma (12/15 reviews, 80.0%).

We separately assessed the 64 reviews from patients where consensus‐level recommendations indicated that there would be no treatment change following review of the classifier results. In this set, additional testing would have been ordered in 25.0% (*n* = 16) of reviews including PD‐L1 (*n* = 7) and HRD (*n* = 6).

Additionally, we found that confirmatory germline testing was recommended for 25.4% (*n* = 105) of the full set of 414 reviews, of which 49.5% (*n* = 52) were reported as being directly based on the results from the molecular diagnostic classifier.

### 3.4 Evaluating confidence in molecular diagnostic classification

While the Tempus TO test and other molecular diagnostic classifiers can provide predicted primary sites for individual patients, a critical component of these predictions is the degree to which clinicians find them diagnostically valuable or believable. To assess this, we asked reviewing oncologists their agreement with the following statement “The TO result increased my confidence in selecting the most appropriate treatment regimen” for each review. Of the 414 reviews, the response breakdown was: Strongly Agree (*n* = 242, 58.5%), Agree (*n* = 130, 31.4%), Neutral (*n* = 31, 7.5%), Disagree (*n* = 10, 2.4%), and Strongly Disagree (*n* = 1, 0.2%; Table 2).

For the 64 reviews from patients where consensus‐level recommendations noted no treatment change following review of the classifier results, 85.9% of reviews from oncologists either strongly agreed (*n* = 36, 56.2%) or agreed (*n* = 19, 29.7%) that the molecular diagnostic classifier results increased their confidence in selecting the most appropriate treatment regimen. Thus, while the classifier results did not lead to a treatment change in these reviews, the results added confidence to the presumed diagnosis and treatment plan.

## 4 DISCUSSION

This retrospective study was designed to assess the potential clinical utility of a molecular diagnostic classifier in informing clinical management decisions for patients who have cancer with an uncertain primary site. For 81.3% of the 289 patients in the study, reviewing oncologists stated that the predicted diagnosis from the molecular diagnostic classifier would result in a change in treatment recommendations.

The results of this study illustrate the potential clinical benefit of performing molecular diagnostic classification in patients where standard‐of‐care diagnostic workups have been unable to render a definitive diagnosis. One explanation for the large rate of change in treatment recommendations that was observed is that treatment regimens for metastatic cancer patients are increasingly based on guidelines (such as the NCCN), which are in turn based on tissue site, histology, and precision medicine biomarkers. Prior studies have assessed the clinical impact of molecular diagnostic classifiers and demonstrated variable rates of treatment impact for CUP patients.^22^ Our study was unique in its attempt to understand the overall impact on treatment management in addition to describing specific types of treatment changes and the need for further biomarker testing, which provides additional details on how molecular diagnostic classifiers can impact clinical management.

When evaluating recommended treatment changes from individual clinical reviews, we observed the addition and removal of essentially all classes of systemic therapies including chemotherapy, tyrosine kinase inhibitors, and CPIs, highlighting the broad spectrum of treatment modalities used in histologic and tissue‐specific regimens. CPIs specifically represent an interesting treatment subset to highlight given the recency of the indications and specificity of guidelines, which are based on histology, site of diagnosis, and PD‐L1 status.^25^ We observed that 19% of reviews would have added CPIs to the patient's treatment regimen, and 6% of reviews would switch the patient from chemotherapy to a CPI alone as their first systemic treatment recommendation. The importance of CPIs was also captured in review‐level assessment with the need for additional biomarker testing: PD‐L1 testing was recommended in the majority (*n* = 118, 58%) of the 203 reviews that specified a requirement for additional testing.

**FIGURE 4:**
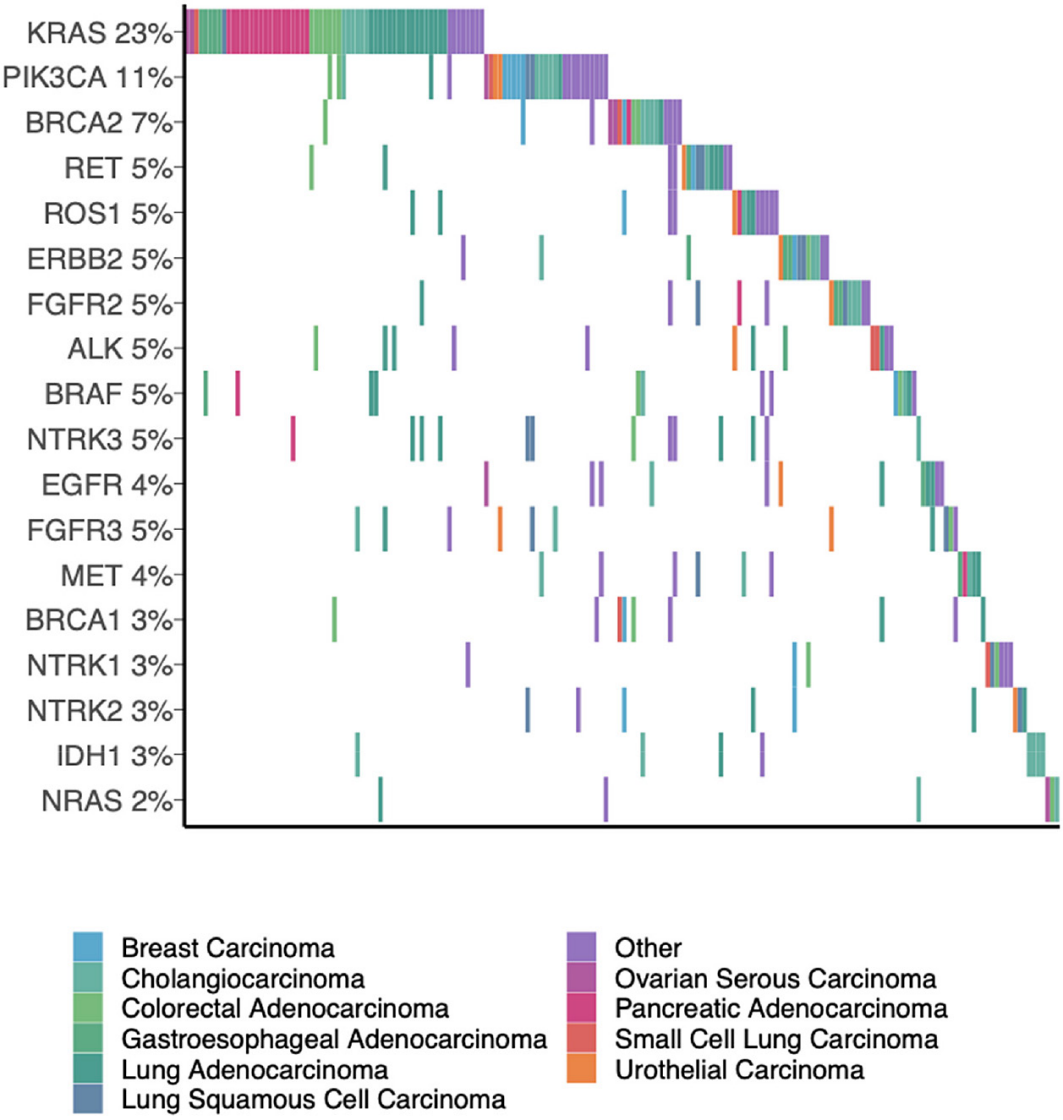
Oncoplot of pathogenic, clinically relevant gene alterations in the study cohort.

While the molecular diagnostic classifier in the study uses transcript expression to predict tumor subtypes, pairing this test with an NGS sequencing panel allowed us to investigate the prevalence of mutations in specific clinically relevant genes and their association with predicted subtypes. Figure 4 highlights the 9 most common subtypes (with the remaining subtypes combined in the “Other” category for the purposes of visualization) and 17 biologically relevant genes corresponding to those subtypes across our 289 patient cohort. Notably, the identified genes highlight additional clinical scenarios where having an accurate diagnosis is important for clinical management decisions. For instance, PARP inhibitors have approval for use in patients with *BRCA1/2* alterations in ovarian, prostate, breast, and pancreatic cancer; however, there is no pan‐cancer *BRCA1/2* approval for these agents.^26^ Additionally, alterations to clinically relevant genes are frequently used as biomarkers for inclusion in clinical trials where knowledge of a tissue‐specific diagnosis may be required for enrollment, such as FIGHT‐101 and BEACON. It should be noted that genomic alterations can also be used to inform the tumor diagnosis such as *IDH* or *FGFR* fusions in patients with liver lesions, indicating a high likelihood of cholangiocarcinoma.^27^ Identification of highly specific genes such as these in specific clinical contexts could potentially obviate the need for the addition of a molecular diagnostic classifier to render or confirm a diagnosis.

Importantly, in the majority of reviews, the molecular diagnostic classifier increased confidence in the therapeutic plan regardless of whether the test results altered treatment recommendations. This confidence is particularly important for cases in the clinical care setting when diagnosis and histology are uncertain. In addition to influencing treatment selection, this confidence is critical in cases with adverse subsequent outcomes. Under these circumstances, the acceptance of subsequent supportive care could be delayed or declined by patients seeking additional opinions and evaluations.

There are several limitations to this analysis. While the primary endpoint of our study was assessed at the patient level and received adjudication by a third oncologist when there was discordance, secondary analyses were performed on review‐level data and did not undergo adjudication. The lack of adjudication for these secondary endpoints is a limiting factor for interpretability of some sub‐analyses given the substantial amount of reviewer discordance observed in these nuanced scenarios of clinical management. Discordance in some of these scenarios is to be expected, as clinical practice heterogeneity may be amplified in the setting of advanced‐stage cancer. In this study, the reviewing oncologists represented a spectrum of oncology specializations and years in practice, which is expected to lead to some degree of clinical management heterogeneity. A further limitation of this survey is that we did not require oncologists to specify a treatment prior to review of the diagnostic classifier results. In clinical practice, even if the ultimate diagnosis is unknown, some form of treatment would be given.

Finally, given that our study is retrospective and de‐identified, we could not evaluate if or how actual clinical management was impacted by the molecular diagnostic classifier results, and instead all of the recommendations that we report are theoretical. Consequently, our study design also does not allow us to determine whether patient outcomes were improved. Future studies aimed at prospectively assessing treatments and outcomes in cohorts of patients diagnosed with CUP are critical to better understand the impact of tissue‐of‐origin classification.

Currently, molecular diagnostic classifiers can support rendering an accurate and actionable cancer diagnosis but are not supported by the NCCN for patients with CUP as indicated in the Occult guidelines.^6^ This position may evolve as precision medicine approaches increasingly depend on tissue and histology diagnoses as observed elsewhere in the NCCN guidelines.^21^ Future prospective and outcome‐based trials are necessary to further demonstrate the clinical utility of molecular diagnostic classifiers for CUP patients.

## AUTHOR CONTRIBUTIONS

**Elle C. Moore:** Conceptualization (equal); data curation (equal); formal analysis (equal); investigation (equal); methodology (equal); project administration (equal); resources (equal); software (equal); supervision (equal); validation (equal); visualization (equal); writing – original draft (equal); writing – review and editing (equal). **Gerard C. Blobe:** Investigation (equal); methodology (equal); resources (equal); writing – review and editing (equal). **Nicholas C. DeVito:** Investigation (equal); methodology (equal); resources (equal); writing – review and editing (equal). **Brent A. Hanks:** Investigation (equal); methodology (equal); resources (equal); writing – review and editing (equal). **Michael R. Harrison:** Investigation (equal); methodology (equal); resources (equal); writing – review and editing (equal). **Christopher J. Hoimes:** Investigation (equal); methodology (equal); resources (equal); writing – review and editing (supporting). **Jingquan Jia:** Investigation (equal); methodology (equal); resources (equal); writing – review and editing (equal). **Michael A. Morse:** Investigation (equal); methodology (equal); resources (equal); writing – review and editing (equal). **Parvathy Jayaprakasan:** Data curation (equal); investigation (equal); methodology (equal); project administration (equal); resources (equal); software (equal); validation (equal); writing – review and editing (equal). **Andrew MacKelfresh:** Data curation (equal); investigation (equal); methodology (equal); project administration (equal); resources (equal); supervision (equal); writing – review and editing (equal). **Hillary Mulder:** Data curation (equal); formal analysis (equal); methodology (equal); software (equal); visualization (equal); writing – review and editing (equal). **Adam J. Hockenberry:** Data curation (equal); project administration (equal); writing – original draft (equal); writing – review and editing (equal). **Alia Zander:** Data curation (equal); formal analysis (equal); methodology (equal); software (equal); visualization (equal); writing – review and editing (equal). **Martin C. Stumpe:** Conceptualization (equal); resources (equal); supervision (equal); writing – review and editing (equal). **Jackson Michuda:** Data curation (equal); methodology (equal); software (equal); writing – review and editing (equal). **Kyle A. Beauchamp:** Data curation (equal); methodology (equal); software (equal); visualization (equal); writing – review and editing (equal). **Eric Perakslis:** Conceptualization (equal); investigation (equal); methodology (equal); supervision (equal); validation (equal); writing – original draft (equal); writing – review and editing (equal). **Timothy Taxter:** Conceptualization (equal); data curation (equal); formal analysis (equal); investigation (equal); methodology (equal); supervision (equal); validation (equal); writing – original draft (equal); writing – review and editing (equal). **Daniel J. George:** Conceptualization (equal); investigation (equal); methodology (equal); resources (equal); supervision (equal); validation (equal); writing – original draft (equal); writing – review and editing (equal).

## FUNDING INFORMATION

This study was funded by Tempus Labs, Inc.

## CONFLICT OF INTEREST STATEMENT

Tempus Labs is a for‐profit company, and individuals with stated affiliations (ECM, AJH, AZ, MCS, JM, KAB, and TT) receive either direct salary and/or are equity shareholders. The Duke University Medical Center oncologists participating in the study were compensated for their review of patient records. The Duke Clinical Research Institute supported project and data management and received compensation for these activities.

## ETHICS STATEMENT

Analyses were performed using de‐identified data under the exemption Pro00042950 granted from the Advarra, Inc. Institutional Review Board (IRB) on April 15, 2020 based on the Department of Health and “45 CFR 46.104(d)(4)”. All methods were carried out in accordance with relevant guidelines and regulations.

## Supporting information


Data S1.

## Data Availability

Data used in the research was collected in a real‐world health care setting and is subject to controlled access for privacy and proprietary reasons. When possible, derived data supporting the findings of this study have been made available within the paper and its Supplementary Figures/Tables.

## References

[1] Binder C , Matthes KL , Korol D , Rohrmann S , Moch H . Cancer of unknown primary—epidemiological trends and relevance of comprehensive genomic profiling. Cancer Med. 2018;7:4814‐4824. 30019510 10.1002/cam4.1689 PMC6144156

[2] Olivier T , Fernandez E , Labidi‐Galy I , et al. Redefining cancer of unknown primary: is precision medicine really shifting the paradigm? Cancer Treat Rev. 2021;97:102204. 33866225 10.1016/j.ctrv.2021.102204

[3] Pavlidis N . Forty years experience of treating cancer of unknown primary. Acta Oncol. 2007;46:592‐601. 17562435 10.1080/02841860701243095

[4] Stella GM , Senetta R , Cassenti A , Ronco M , Cassoni P . Cancers of unknown primary origin: current perspectives and future therapeutic strategies. J Transl Med. 2012;10:12. 22272606 10.1186/1479-5876-10-12 PMC3315427

[5] Ettinger DS , Agulnik M , Cates JMM , et al. Occult primary. J Natl Compr Canc Netw. 2011;9:1358‐1395. 22157556 10.6004/jnccn.2011.0117

[6] National Comprehensive Cancer Network . Occult Primary (Version 1.2023) [Internet]. Accessed August 2, 2022. https://www.nccn.org/professionals/physician_gls/pdf/occult.pdf

[7] Kamposioras K , Pentheroudakis G , Pavlidis N . Exploring the biology of cancer of unknown primary: breakthroughs and drawbacks. Eur J Clin Invest. 2013;43:491‐500. 23480555 10.1111/eci.12062

[8] Varadhachary G . New strategies for carcinoma of unknown primary: the role of tissue‐of‐origin molecular profiling. Clin Cancer Res. 2013;19:4027‐4033. 23519898 10.1158/1078-0432.CCR-12-3030

[9] Economopoulou P , Mountzios G , Pavlidis N , Pentheroudakis G . Cancer of unknown primary origin in the genomic era: elucidating the dark box of cancer. Cancer Treat Rev. 2015;41:598‐604. 26033502 10.1016/j.ctrv.2015.05.010

[10] Abraham J , Heimberger AB , Marshall J , et al. Machine learning analysis using 77,044 genomic and transcriptomic profiles to accurately predict tumor type. Transl Oncol. 2021;14:101016. 33465745 10.1016/j.tranon.2021.101016 PMC7815805

[11] Michuda J , Breschi A , Kapilivsky J , et al. Validation of a transcriptome‐based assay for classifying cancers of unknown primary origin. Mol Diagn Ther. 2023;27:499‐511. doi:10.1007/s40291-023-00650-5 37099070 PMC10300170

[12] Conway A‐M , Mitchell C , Kilgour E , Brady G , Dive C , Cook N . Molecular characterisation and liquid biomarkers in Carcinoma of Unknown Primary (CUP): taking the “U” out of “CUP.”. Br J Cancer. 2019;120:141‐153. 30580378 10.1038/s41416-018-0332-2 PMC6342985

[13] Hayashi H , Kurata T , Takiguchi Y , et al. Randomized phase II trial comparing site‐specific treatment based on gene expression profiling with carboplatin and paclitaxel for patients with cancer of unknown primary site. J Clin Oncol. 2019;37:570‐579. 30653423 10.1200/JCO.18.00771

[14] Fizazi K , Maillard A , Penel N , et al. A phase III trial of empiric chemotherapy with cisplatin and gemcitabine or systemic treatment tailored by molecular gene expression analysis in patients with carcinomas of an unknown primary (CUP) site (GEFCAPI 04). Ann Oncol. 2019;30:v851.

[15] Moran S , Martínez‐Cardús A , Sayols S , et al. Epigenetic profiling to classify cancer of unknown primary: a multicentre, retrospective analysis. Lancet Oncol. 2016;17:1386‐1395. 27575023 10.1016/S1470-2045(16)30297-2

[16] Varadhachary GR , Talantov D , Raber MN , et al. Molecular profiling of carcinoma of unknown primary and correlation with clinical evaluation. J Clin Oncol. 2008;26:4442‐4448. 18802157 10.1200/JCO.2007.14.4378

[17] Hainsworth JD , Schnabel CA , Erlander MG , Haines DW III , Greco FA . A retrospective study of treatment outcomes in patients with carcinoma of unknown primary site and a colorectal cancer molecular profile. Clin Colorectal Cancer. 2012;11:112‐118. 22000811 10.1016/j.clcc.2011.08.001

[18] Hayashi H , Takiguchi Y , Minami H , et al. Site‐specific and targeted therapy based on molecular profiling by next‐generation sequencing for cancer of unknown primary site: a nonrandomized phase 2 clinical trial. JAMA Oncol. 2020;6:1931‐1938. 33057591 10.1001/jamaoncol.2020.4643 PMC7563669

[19] Yoon HH , Foster NR , Meyers JP , et al. Gene expression profiling identifies responsive patients with cancer of unknown primary treated with carboplatin, paclitaxel, and everolimus: NCCTG N0871 (alliance). Ann Oncol. 2016;27:339‐344. 26578722 10.1093/annonc/mdv543 PMC4907341

[20] Hainsworth JD , Rubin MS , Spigel DR , et al. Molecular gene expression profiling to predict the tissue of origin and direct site‐specific therapy in patients with carcinoma of unknown primary site: a prospective trial of the Sarah Cannon research institute. J Clin Oncol. 2013;31:217‐223. 23032625 10.1200/JCO.2012.43.3755

[21] NCCN . Treatment by Cancer Type [Internet]. Accessed August 4, 2022. https://www.nccn.org/guidelines/category_1

[22] Thomas SP , Jacobson LE , Victorio AR , et al. Multi‐institutional, prospective clinical utility study evaluating the impact of the 92‐gene assay (CancerTYPE ID) on final diagnosis and treatment planning in patients with metastatic cancer with an unknown or unclear diagnosis. JCO Precis Oncol. 2018;2:1‐12. 10.1200/PO.17.00145 35135112

[23] Beaubier N , Bontrager M , Huether R , et al. Integrated genomic profiling expands clinical options for patients with cancer. Nat Biotechnol. 2019;37:1351‐1360. 31570899 10.1038/s41587-019-0259-z

[24] Beaubier N , Tell R , Lau D , et al. Clinical validation of the Tempus xT next‐generation targeted oncology sequencing assay. Oncotarget. 2019;10:2384‐2396. 31040929 10.18632/oncotarget.26797 PMC6481324

[25] Approval Timelines of Active Immunotherapies [Internet] . Cancer Research Institute. Accessed September 19, 2022. https://www.cancerresearch.org/es/scientists/immuno‐oncology‐landscape/fda‐approval‐timeline‐of‐active‐immunotherapies

[26] Rose M , Burgess JT , O'Byrne K , Richard DJ , Bolderson E . PARP inhibitors: clinical relevance, mechanisms of action and tumor resistance. Front Cell Dev Biol. 2020;8:564601. 33015058 10.3389/fcell.2020.564601 PMC7509090

[27] Saha SK , Zhu AX , Fuchs CS , Brooks GA . Forty‐year trends in Cholangiocarcinoma incidence in the U.S.: intrahepatic disease on the rise. Oncologist. 2016;21:594‐599. 27000463 10.1634/theoncologist.2015-0446 PMC4861366

