# Supplementary material for: Assessing the utility of molecular diagnostic classification for cancers of unknown primary
Source: Cancer Med. 2023 Sep 15;12(19):19394–405. doi: 10.1002/cam4.6532 (PMC10587948; doi:10.1002/cam4.6532)
Supplement: Supplementary file 1 — Data S1. [file CAM4-12-19394-s001.pdf]

Use the "TO-### Clinical Information" pdf only to answer this portion of the assessment

1. Name of Reviewer
   dropdown

2. Patient Study ID
   validated free-text

3. Does the patient's cancer appear to be of uncertain/unknown primary?
   single select
   - Yes - Proceed to question 4
   - No - Skip to question 7
   - Not enough information- Skip to question 15

4. How many organs/cancer types are included in your differential diagnosis?
   single select
   - 2
   - 3
   - 4
   - 5+

5. What treatment regimen would you give, based on the patient's clinical information?
   free text

6. What type of treatment is this? Select all that apply.
   multi select
   {Skip to question 8 after this question is answered}

   - CUP guidelines-guided
   - Molecular-guided
   - N/A - Not enough information to select a treatment
   - Other (Opens free text)

7. What information influenced your decision to classify this patient as not CUP? Select all that apply.
   {Skip to question 15 after this question is answered}
   multi select

   - Pathology/IHC

- Imaging
- Clinical History
- Other (opens free text)

Review the "TO-XXX Tumor Origin Report" pdf ahead for this section of the study assessment

8. Is additional organ-specific testing needed to make a treatment decision, based on the Tumor Origin test results? Select all that apply.
   multi select

   - No - Skip to question 10
   - PD-L1 – Proceed to question 9
   - HRD – Proceed to question 9
   - ER/PR/HER2 – Proceed to question 9
   - Additional imaging- Proceed to question 9
   - Other (opens free text) – Proceed to question 9

9. How would the results of additional organ-specific testing change treatment?
   {Skip to question 13 after this question is answered}
   single-select

   - Treatment would change regardless of the results of testing
   - Treatment change is dependent on result of testing
   - Treatment would not change
   - Other (opens free text)

10. What treatment regimen would you give, based on the Tumor Origin test results in conjunction with the clinical information?
    free text

11. What type of treatment is this? Select all that apply.
    multi select

    - CUP guidelines-guided
    - Molecular-guided
    - Organ of origin-specific

- Not enough information to select a treatment

12. Did the treatment regimen change?
    single select

    - Yes, the entire regimen was updated
    - Yes, I added an agent
    - Yes, I removed an agent
    - No
    - N/A - Not enough information to select a treatment
    - Other (opens free text)

13. The TO result increased my confidence in selecting the most appropriate treatment regimen.
    single select

    - Strongly Agree
    - Agree
    - Neutral
    - Disagree
    - Strongly Disagree

14. Would you order confirmatory germline testing? Select all that apply.
    {Skip to question 16 after this question is answered}
    multi select

    - Yes, based on the Tumor Origin test results
    - Yes, based on family history
    - Yes, based on previous germline testing
    - No
    - Not enough information to determine

15. Would the result of the TO test change your management of the patient? Select all that apply.
    multi select

    - No
    - Treatment would change
    - Additional workup
    - Other

16. Comments for study personnel or data analysis (Optional)

free text
